# p53 Mutation as Plausible Predictor for Endocrine Resistance Therapy in Luminal Breast Cancer

**DOI:** 10.12688/f1000research.108628.2

**Published:** 2022-11-30

**Authors:** Freda Halim, Yohana Azhar, Suwarman Suwarman, Bethy Hernowo

**Affiliations:** 1 Department of Surgery, Pelita Harapan University, Tangerang, Indonesia; 2 Department of Surgery - Oncology, Head and Neck Division, Hasan Sadikin General Hospital, Universitas Padjajaran, Bandung, Indonesia; 3 Department of Anesthesiology and Intensive Care, Hasan Sadikin General Hospital, Universitas Padjajaran, Bandung, Indonesia; 4 Department of Anatomical Pathology, Universitas Padjajaran, Bandung, West Java, Indonesia

**Keywords:** p53, predictor, endocrine therapy resistance, luminal breast cancer

## Abstract

Endocrine therapy resistance in Luminal Breast Cancer is a significant issue to be tackled, but currently, no specific biomarker could be used to anticipate this event. p53 mutation is widely known as one of Breast Cancer’s most prominent genetic alterations. Its mutation could generate various effects in Estrogen Receptor and Progesterone Receptor molecular works, tangled in events leading to the aggravation of endocrine therapy resistance. Hence the possibility of p53 mutation utilization as an endocrine therapy resistance predictive biomarker is plausible. The purpose of this review is to explore the latest knowledge of p53 role in Estrogen Receptor and Progesterone Receptor molecular actions, thus aggravating the Endocrine Therapy resistance in Luminal Breast Cancer, from which we could define possibilities and limitations to utilize p53 as the predictive biomarker of endocrine therapy resistance in Luminal Breast Cancer.

## Introduction

Endocrine Therapy (ET) resistance in Luminal Breast Cancer (BC) is a concerning issue. Approximately 30-40% of Luminal BC are ET resistant, which leads to a higher recurrence rate and worsened prognosis. Although it has been extensively studied, till now there is no single predictive biomarker has been established to predict which patient will develop ET resistance during the 5-years-course of endocrine therapy. ^1^ ^–^ ^3^

Such predictive biomarkers will be advantageous for clinicians and patients, as patients with a bigger chance of endocrine therapy resistance could be monitored closely. Perhaps later in the future, it could help to effectively change the course of the therapy before recurrence is established (and it becomes too late), as well as to help clinicians to identify which patients will not have ET benefits in the first place. ^1^ ^,^ ^2^ As we know, the current trend in clinical trials of BC treatment is moving into personalized and tailored therapy for each case. Therefore, finding predictive biomarkers to predict ET resistance will also be critical for such therapeutic program development. ^3^

Endocrine therapy resistance is a complex molecular process involving many development processes. Several hypotheses have been developed regarding addressing such a process and finding such predictive biomarkers. The resistance could develop at the start of the endocrine therapy ( *de novo* or intrinsic resistance) or develop later during the endocrine therapy. The hypotheses range from the loss of hormonal receptor (HR) caused by *ESR1* gene mutation and epigenetic mechanism, ^4^ ^–^ ^7^ altered expression of co-factors (such as NF-kB, AIB1, SRC-1), ^8^ ^–^ ^10^ crosstalk between ER and growth factors signaling (such as Her2neu, Insulin-like growth factor-1 receptor (IGF-1R)) ^3^ ^,^ ^5^ ^,^ ^7^ ^,^ ^9^ ^,^ ^11^ ^,^ ^12^ absent or reduced expression of a negative regulator such as p21 and p27, ^13^ ^–^ ^15^ metabolic resistance caused by polymorphism or loss of CYP2D6 (main enzymes responsible for converting tamoxifen into its active metabolites), ^2^ ^,^ ^3^ ^,^ ^7^ ^,^ ^9^ ^,^ ^16^ ^,^ ^17^ *NF1* mutation lead to MAPK pathway activation, ^18^ ^–^ ^23^ APOBEC mutation associated with *PI3KCA* mutation. ^24^

The molecular mechanism of Estrogen Receptor (ER) and Progesterone Receptor (PR) actions are studied extensively for their association with ET resistance in Luminal BC. These molecular mechanisms additionally become an essential basis in rationalizing treatments such as Cyclin-CDK (Cyclin-Dependent Kinase) inhibitor and PI3K/Akt/mTOR inhibitor, which have been internationally accepted as current adjuvant treatments for Luminal BC with recurrence after ET resistance. Their actions, therefore, are fundamental knowledge to find a logical explanation of endocrine therapy resistance, and most of the hypotheses above could be explained by the disruption of the ER and PR mechanism of actions, resulting increased cellular proliferation and decreased apoptosis. ^3^ ^,^ ^5^ ^,^ ^7^ ^–^ ^15^ ^,^ ^25^ ^,^ ^26^

p53 mutation is one of the most frequent genetic alterations in BC, found in approximately 28.3%-35% of overall BC patients, with higher incidence in Luminal B BC (30-55%), Her-2neu overexpression (70%) and TNBC group (80%). ^27^ ^–^ ^29^ p53 mutation in positive hormonal BC will result in distinct poor prognosis, and especially seen in Luminal B BC with higher frequency and stronger association to poor prognosis compared to Luminal A BC. ^28^ ^,^ ^30^ The mutation of this profound tumor suppressor gene may occur at the early onset of Luminal BC or progressively in the later course of the disease due to cancer cells’ ability to form more mutations in the advanced stage. ^19^ ^,^ ^27^ ^,^ ^31^ ^–^ ^33^

p53 mutation has been known for more than four decades. Its extensive roles span cell cycle regulation, DNA repair, apoptosis process, cell metabolism, and immune response in the tumor microenvironment. ^20^ ^,^ ^33^ ^–^ ^37^ This versatile tumor suppressor gene has been studied in many cancers, including breast cancer. Numerous endocrine resistance breast cancer studies conclusively found its protein accumulation and its mutation. ^19^ ^,^ ^31^ ^,^ ^32^ ^,^ ^38^ ^,^ ^39^

This review will explore the current knowledge of ER and PR molecular mechanisms and their impact on initiating ET resistance in Luminal BC. Furthermore, we will discuss the apparent effect of p53 mutation on their molecular mechanisms, consequently aggravating ET resistance.

## Estrogen and estrogen receptor

Estrogen is a steroid hormone in several tissues, such as the skin, liver, bone, and breast. Estrogen’s potent mitogenic effect in breast tissue will generate breast epithelial proliferation, alveolar growth, fat deposition, and fibrous tissue development during puberty, pregnancy, and lactation phases. These unprecedented changes in the breast are affected by Estrogen, which works alongside Progesterone and other growth factors. ^40^

The active form of Estrogen in breast tissue, Estradiol, and its metabolites have been acknowledged as essential factors of early malignant transformation, such as DNA single-strand breaks and chromosomal impairment. Furthermore, it may lead to uncontrolled cell proliferation, accompanied by the development of cellular signaling collaborating in the cancerous cells’ progression. All events mentioned above will benefit the growth of cancer cells, and all depend on the molecular mechanism of ER in BC cells. ^41^ ^,^ ^42^

## Estrogen receptor and its classical mechanism of action

Estrogen Receptor has a paramount role in BC cells, as described above. Hence it becomes the main target of endocrine therapy such as ovarian blockade, SERM (Selective Estrogen Receptor Modulator, i.e., Tamoxifen), and SERD (Selective Estrogen Receptor Degrader, i.e., Fulvestran). ^9^ ^,^ ^21^

Being a nuclear receptor family member, ER-α and ER-β are the two different types of Estrogen Receptors. In breast tissue, the ER-α has a dominant role. Meanwhile, ER-β is still considered controversial and has an unclear role. ^43^ Another estrogen receptor type is the *G-coupled Estrogen Receptor* (GPER), paramount for estrogen molecular action via the membranous mechanism. ^44^

ER-α coded by the *ESR-1* gene in chromosome 14, with an identical structure as other nuclear receptors, consists of 4 structural and functional domains. These domains are the amino-terminal domain (A/B domain), DNA binding domain/DBD, hinge region (D domain), and Ligand-Binding Domain/LBD. ^45^

While estrogen binds with ER, the heat shock proteins (HSP70 and HSP90) will dissociate ER from this binding in the cytosol. This dissociation will cause conformation changes and form dimers, then ER-Estrogen dimers will be transported to the nucleus by D-domain, and subsequently the dimers will form attachment to EREs. ^9^ ^,^ ^25^ ^,^ ^46^ ^,^ ^47^

Subsequently, after entering the nucleus, DBD, with the aid of co-activators, will bind to Estrogen’s target genes that contain Estrogen Response Elements (EREs). The known co-activators are steroid receptor co-activator-1/SRC-1, SRC-2, and SRC-3 (AIB1/Amplified in Breast-Cancer 1). The binding of ER and EREs will activate the transcription of Estrogen’s target genes. ^3^ ^,^ ^48^ ^,^ ^49^ This process is the so-called classic mechanism of ER molecular action, depicted in the figure below, along with other mechanisms. This mechanism is the first known ER molecular action and has become the theoretical basis for applying traditional endocrine therapy in luminal BC, such as ovarian blockade, SERM, and SERD. ^9^ ^,^ ^23^

## Other molecular actions of estrogen receptor

**Figure 1.**
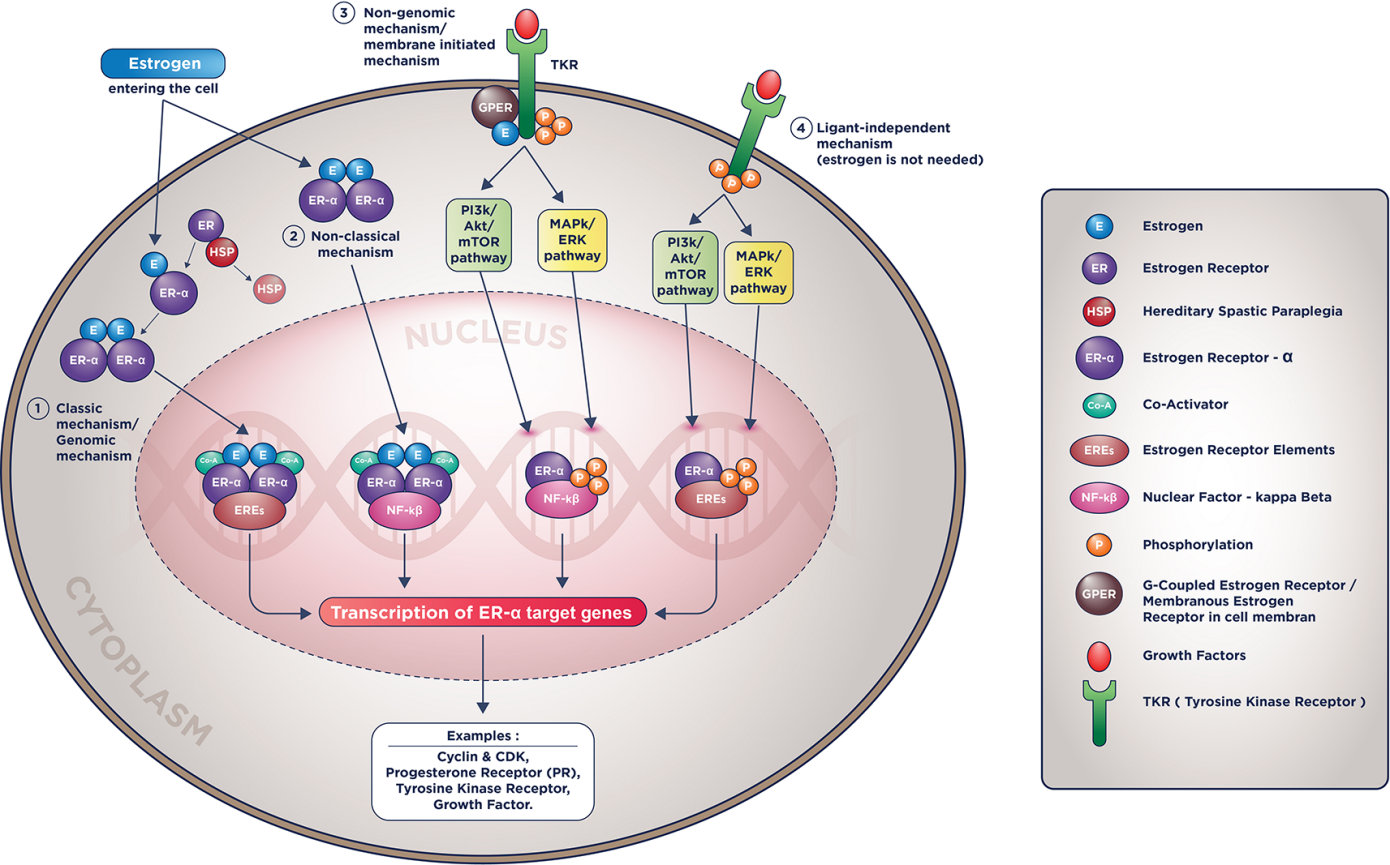
ER-α molecular mechanisms of action.

The estrogen receptor molecular actions are complicated and involve the intersecting apoptotic along with the survival pathways such as PI3K/Akt/mTOR, MAPK/ERK, resulting in the same similar target genes such as Cyclin-CDK, growth factor, and its activators. ^9^ ^,^ ^50^ Currently, there are four known molecular mechanisms of ER actions: classical (genomic), non-classical, non-genomic (membranous), and ligand-independent (estrogen-independent). These four mechanisms are pictured in Figure 1.

In the non-classical mechanism (2 ^nd^ mechanism in Figure 1), Estrogen could activate genes transcription that doesn’t contain EREs with help from tethering co-factors such as NF-kB (Nuclear Factor-Kappa Beta), activator protein 1 (AP-1), or specificity protein 1 (SP-1). ^3^ ^,^ ^9^

In the membranous mechanism, Estrogen will not be required to enter the nucleus to do the genomic action since ER receptors conduct all the inciting processes in the cell membrane. In the last mechanism, even Estrogen is not required to induce its target genes' transcription (hence the name ligand-independent mechanism). ^3^ ^,^ ^9^

These mechanisms will induce the exact effect on breast cancer cells: accentuating proliferative pathways and diminishing pro-apoptotic pathways. These non-classic, membranous, and particularly ligand-independent mechanisms make cancer cells more resistant to endocrine therapy. It is as if these estrogen receptors’ mechanism of action is being “hijacked” by the cells; the cancer cells are manipulating it to their benefit, that is, to replicate more and become less sensitive to apoptotic signals. ^4^ ^,^ ^9^ ^,^ ^21^ ^,^ ^44^

The result of these molecular mechanisms of ER receptor actions are constant activation of the estrogen receptor target genes although there were no more estrogen molecules available ( i.e., due to ovarian blockade or inhibition by aromatase inhibitor), and although its receptor being blocked or degraded (i.e., due to inhibition by SERM/SERD). ^4^ ^,^ ^9^ ^,^ ^21^ ^,^ ^44^ Despite this established ER receptor actions, we acknowledged that ET resistance is multifactorial and we cannot exclude several other non-estrogen related pathways in several studies using tamoxifen and fulvestrant adapted cell lines. ^51^ ^–^ ^54^

## Progesterone and progesterone receptor

Progesterone is a steroid hormone produced by the corpus luteum in the human ovarium, which its primary duty is to prepare the female body for gestation. Breast epithelial cells are indispensable in affecting duct-alveolar changes in phases such as puberty, the luteal phase (pre-menstrual period), pregnancy, and lactation. ^55^

Compared to Estrogen, the role of Progesterone in breast cancer cells, especially endocrine therapy, and its resistance is less distinctive and less studied. The cyclic level of Progesterone and its hundreds of active metabolites available in the female body are the main difficulties in testing this hormone. ^56^ *PGR* and *ESR1* are usually co-expressed and only minor cases described *PGR* expression alone. ^57^ Furthermore, the target genes of ER and PR overlap, thus adding to the complexity of this issue. ^56^ Still, as epidemiological observations have shown, one cannot ignore the fact that the combination of Progesterone and Estrogen will add a mitogenic effect to BC cells in the animal model. ^58^ ^–^ ^60^

PR was transcribed by three means. First, its transcription is induced by Estrogen as *PGR* (the gene for encoding the PR) is one of the Estrogen target genes. Estrogen has been proven to be required in maintaining PR levels in breast and endometrium epithelial cells. ^61^ Second, cancer cells could induce PR transcription mediated by Insulin Growth Factor-1 (IGF-1) and MAPK/ERK activity. Even more, at high Progestin concentration, these growth factors will be re-induced and thus will re-activate the ER-α phosphorylation in the ligand-independent mechanism of ER (review above figure), resulting in more PR transcription. ^62^

Some of the Progesterone target genes are also known to overlap with Estrogen target genes such as Cyclin-CDK, RANKL (Receptor activator of nuclear factor-kappa-Β ligand), and other growth factors. Hence these crosstalk mechanisms between ER and PR are crucial for breast cancer cell carcinogenesis and endocrine therapy resistance. ^4^ ^,^ ^62^

Like Estrogen, the action of Progesterone in cells is entirely dependent on its receptors, and it consists of both nuclear and membranous receptor types. There are two types of nuclear PR: PR-A and PR-B. Both nuclear receptors exist in breast epithelial cells in variable amounts and activity. Furthermore, which nuclear receptor is more dominant in breast epithelial cells is unclear. ^56^

Identical to ER, PR has an N-terminal domain, Ligand Binding Domain/LB, Progesterone will bind the PR and DNA Binding Domain/DBD in which target genes contain *Progesterone Receptor Elements* (PREs) will bind. ^63^

**Figure 2.**
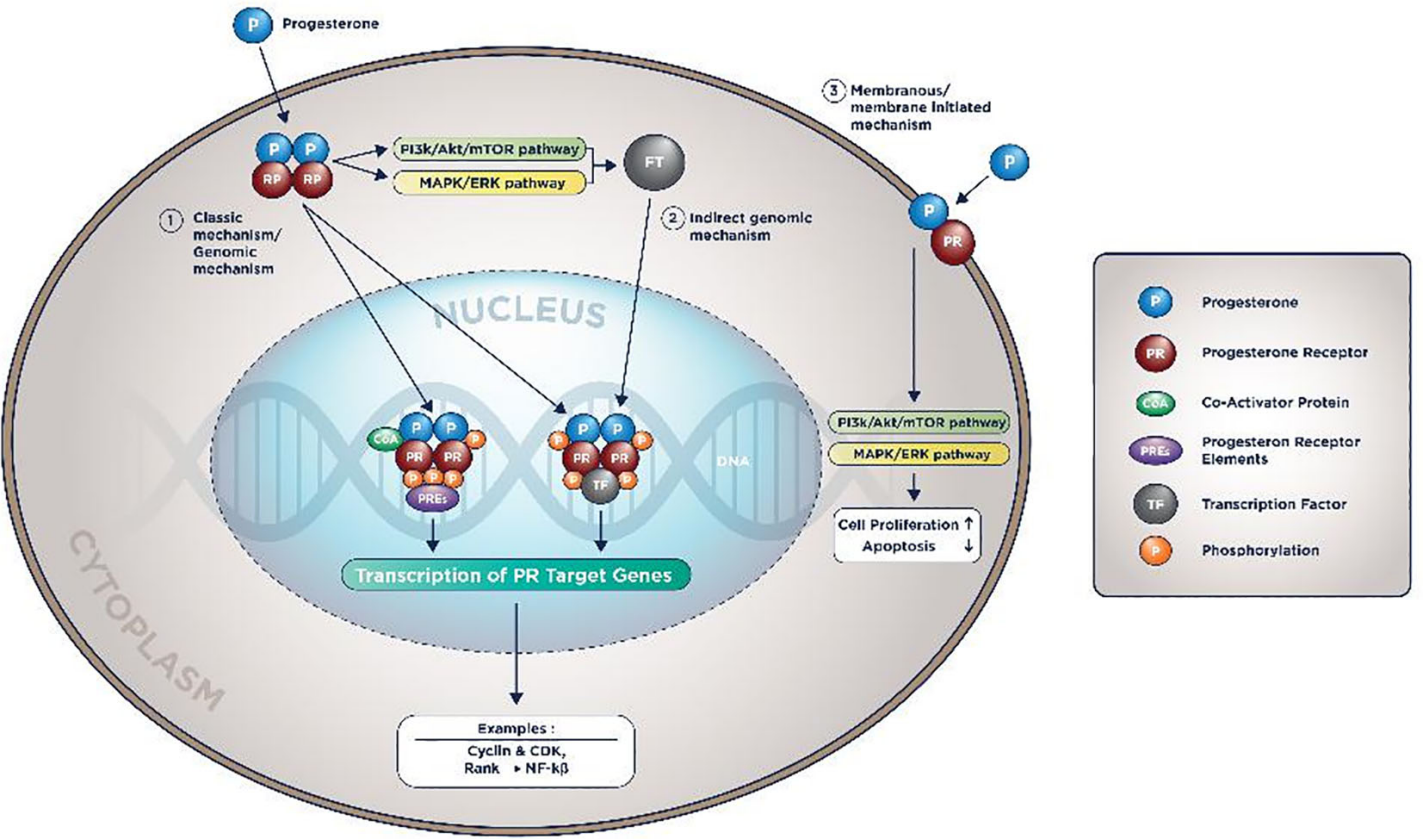
PR molecular mechanism of action.

After entering the cell’s cytoplasm, Progesterone will form a dimer, bind to PR in LBD, enter the nucleus, and bind to PREs with a co-factor. This process will activate the transcription of PR target genes. This is known as the PR action’s classical/direct genomic mechanism. ^56^ Other mechanisms known are the non-classical/direct non-genomic and membranous mechanisms, depicted in Figure 2. ^15^ ^,^ ^56^

In the indirect genomic mechanism, progesterone could activate genes that do not contain PREs as long there are tethering co-factors. In the membranous mechanism, PI3K/Akt/mTOR pathway and MAPK/ERK are also activated by progesterone. Therefore, it will accentuate the proliferative pathways and diminish the pro-apoptotic signals. ^15^ ^,^ ^56^

## p53 involvement in pathways activated in ER and PR molecular of actions

### p53 in cell cycle regulation

The complicated cellular signaling regulates the cell cycle to maintain the regular cell proliferation rate and minimize errors in DNA synthesis. In this cell cycle, abnormal cells with DNA error will be ceased in G1-S transition critical point. ^64^

Essentially, this critical G1-S transition point is determined by the interaction of Cyclin-D1& CDK4/6. This interaction will release E2F protein from its bond with Retinoblastoma Protein (RB Protein) in conditions without inhibition. The E2F protein will further trigger the cell to enter the S phase. Then consequently, abnormal cells with DNA will be duplicated. ^65^

This mechanistic complex is one of the most often disrupted cellular signaling. It is found in endocrine therapy-resistant breast cancer cells, as Cyclin D1 (CCND1) and CDK4 become the target genes of Estrogen and Progesterone. Previously, Cyclin-CDK is still transcribed by the cancer cells, although the Estrogen production has been diminished and their receptors have been blocked. ^21^ ^,^ ^48^ ^,^ ^49^ Relevantly, CDK 4/6 inhibitor has been approved in clinical guidelines as an adjunctive for endocrine therapy in Luminal BC, both pre-and postmenopausal patients. ^12^ ^,^ ^66^

In normal cellular regulation, cells with abnormal DNA will be forced to enter the G0 phase by the p21 protein, a protein transcribed and regulated by p53. This p21 protein will inhibit the CyclinD1-CDK4/6 complex, resulting in the cell entering the G0 phase and starting the DNA repairing process. This well-regulated system earned p53 the old nickname: “guardian of the genome”. ^67^ ^,^ ^68^

## p53 involvement in PI3K/Akt/mTOR pathway

PI3K/Akt/mTOR pathway is a series of consecutive intracellular signaling that will activate proliferation and prevent apoptotic events. Genetic accumulation in this pathway and mutation of its inhibitor (PTEN/Phosphatase and TENsin homolog deleted on chromosome 10) are found in about 70% of the whole BC population. ^11^ ^,^ ^69^

PI3K is an intracellular lipid kinase enzyme that will phosphorylate phosphatidylinositol molecule in the cell membrane, turning phosphatidylinositol-4,5-bisphosphate (PIP2) into phosphatidylinositol-3,4,5-trisphosphate (PIP3). ^69^ Afterward, PIP3 will facilitate interaction between phosphoinositide-dependent kinase 1 (PDK-1) and Akt in the cell cytoplasm, resulting in a phosphorylated Akt. This phosphorylated Akt will activate Forkhead box O transcription factor (FoxO), which inhibits pro-apoptosis genes and activates mechanistic targets of rapamycin (mTOR) complexes. ^11^

The mTOR complexes consist of 2 active forms: activated mTORC1 and mTORC2. mTORC1 will activate genes involved in carcinogeneses like protein synthesis, pro-survival genes, and cell growth. mTORC2 will specifically enhance phosphorylation, further causing Akt hyperactivation. ^11^ ^,^ ^69^

In ER-α molecular actions, PI3K/Akt/mTOR will be activated in the non-classical, membranous, and ligand-independent mechanism. ^9^ ^,^ ^44^ ^,^ ^50^ ^,^ ^70^ A likely, PI3K/Akt/mTOR will also be activated in PR molecular actions. ^15^ ^,^ ^56^ Additionally, mTOR1 will activate S6K, which will help to phosphorylate RE-α, further activating the functional domain of RE-α. Likewise, the Akt activates the NF-kB that functions as a co-factor in the non-classical and membranous mechanism of ER-α molecular actions. ^20^

It is well known that PTEN, a classical tumor suppressor gene, will reverse PIP3 to PIP2; hence the subsequent Akt/mTOR activation will not occur. Without PTEN, PI3K/Akt/mTOR pathway will be hyperactivated. ^71^

The wild-type p53 protein will activate *PTEN* gene transcription. In cells with mutant p53, *PTEN* gene mRNA expression will be drastically reduced compared to cells with wild-type p53 status. ^72^ Another seminal finding by Jung *et al.* 2018 in cell culture studies shows cells with PTEN loss will cause PI3K/Akt/mTOR hyperactivation, causing mTORC1 and mTORC2 enhancement. Both will phosphorylate and activate wild-type p53 protein, which causes p21 transcription. p21 protein will further induce cells to premature senescence condition. ^73^

## p53 roles in tumor microenvironment in ET resistant BC and NF-kB pathway

Although Luminal BC is considered ‘cold tumor’ due to its low immunogenicity characterized by the low count of Tumor Infiltrating Lymphocytes (TILs) and low Programmed Death Ligand-1 receptor, the roles of tumor microenvironment in ET resistance progression could not be ignored. ^74^ ^–^ ^79^ Recent study by Gomez, *et al.* 2020 showed that exposure of ER+HER2-cells to continuous RANK pathway (a member of the tumor necrosis factor receptor (TNFR) superfamily) activation by exogenous RANKL (receptor activator of nuclear factor-kB ligand) both in vitro and in vivo, will cause downregulation of HR and increased resistance to hormone therapy. ^11^ Sobral-leite, *et al.* 2019 depicted that activation of the PI3K pathway, in breast tumor cells was positively correlated with tumor-infiltrating FOXP3-positive lymphocytes which will bring poor prognosis for the patients. ^80^ Another seminal study by Anurag, *et al.* 2020 identify that Luminal B BC have significantly high immunologic properties gene expressions correlated with endocrine resistant BC such as *IDO1, PD1, LAG3* which will induce cytotoxic T-cell tolerance and down regulation of T-cell activation, and those three genes are targetable for immune-checkpoint inhibitor. ^76^

The roles of p53 in BC tumor microenvironment is also noted, among which of the most discussed is p53 and its association with NF-kB. *Nuclear Factor-kappa Beta* (NFKB) is a transcription factor family consisting of 5 subtypes: p50, p52, p65 (RelA), RelB, and c-Rel. With a vast target gene involved in chronic inflammation and cellular proliferation, NF-kB is studied extensively in many cancers, including endocrine-resistant Luminal BC. In cell culture studies, treatment with NF-kB inhibitors will evoke endocrine therapy sensitivity and toxicity; therefore, it has not been tested on humans. ^8^

NF-kB target genes considered instrumental in endocrine therapy resistance evolution are Cyclin D1, D2, D3 dan E, anti-apoptotic protein Bcl-2, MDM-2, and PDL-1 ( *Programmed Death Ligand-1*). ^8^ ^,^ ^25^ ^,^ ^81^ In ER molecular actions explained above; the NF-KB works as a co-factor in non-classical and membranous mechanisms so that Estrogen’s target genes are still transcribed although Estrogen has been blocked. ^8^ ^,^ ^78^ ^,^ ^81^ ^–^ ^84^

The p53 and NF-kB have a negative association, for one cannot exist if the others are activated. The function is also contradictive; NF-kB will cause cell proliferation, be anti-apoptotic, and enhance chronic inflammation, whereas p53 will regulate the cell cycle and trigger pro-apoptotic events when needed. ^81^ ^,^ ^85^

The antagonistic mechanisms are various. Some studies noted wild-type p53 protein act as the direct promoter inhibitor of NF-kB target genes, therefore inhibiting the transcription of the NF-kB target genes. Others stated they compete with each other to get transcription co-factor 300. Wild-type p53 protein will also be known to inhibit the IKK enzyme (Inhibitor of Kappa Kinase, an enzyme to activate the active form of NF-kB). Without IKK, NF-kB couldn’t enter the nucleus and induce its target genes transcription. ^35^ ^,^ ^81^ ^,^ ^85^

TNF-α, the multi-functional mediator in inflammation and cell apoptosis, is also noted in Luminal BC for its role in enhancing cellular proliferation via NF-kB activation. ^86^ ^,^ ^87^ The wild-type p53 was also recognized in turning off TNF-α induced NF-kB activation. This action is achieved by binding and blocking the work of Disabled homolog 2-interacting protein (DAB2IP), a protein that will activate TNF-α to trigger NF-kB activation. ^86^

**Figure 3.**
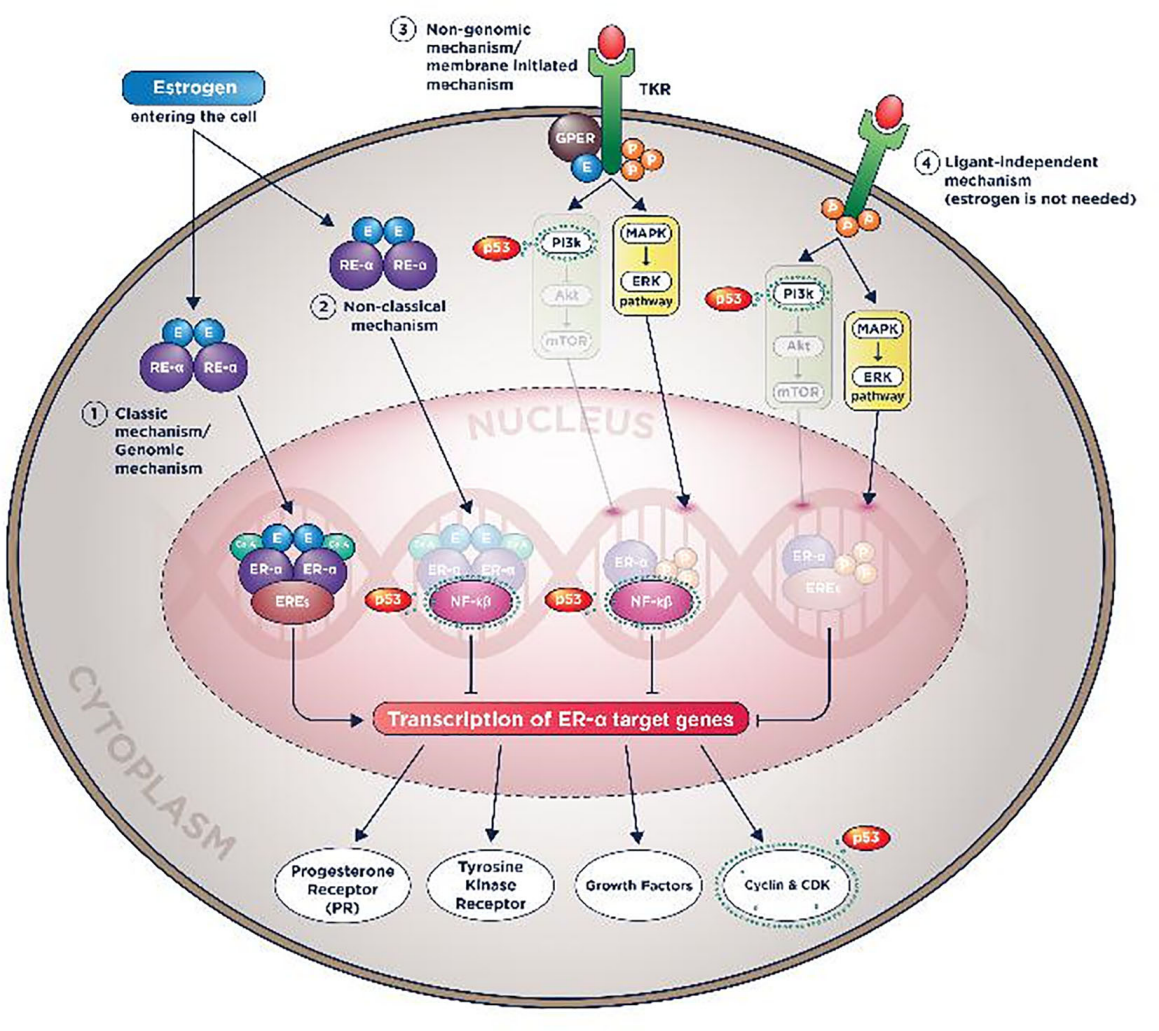
Plausible p53 wild-type roles in reducing endocrine therapy resistance by prohibiting ER-α molecular actions.

**Figure 4.**
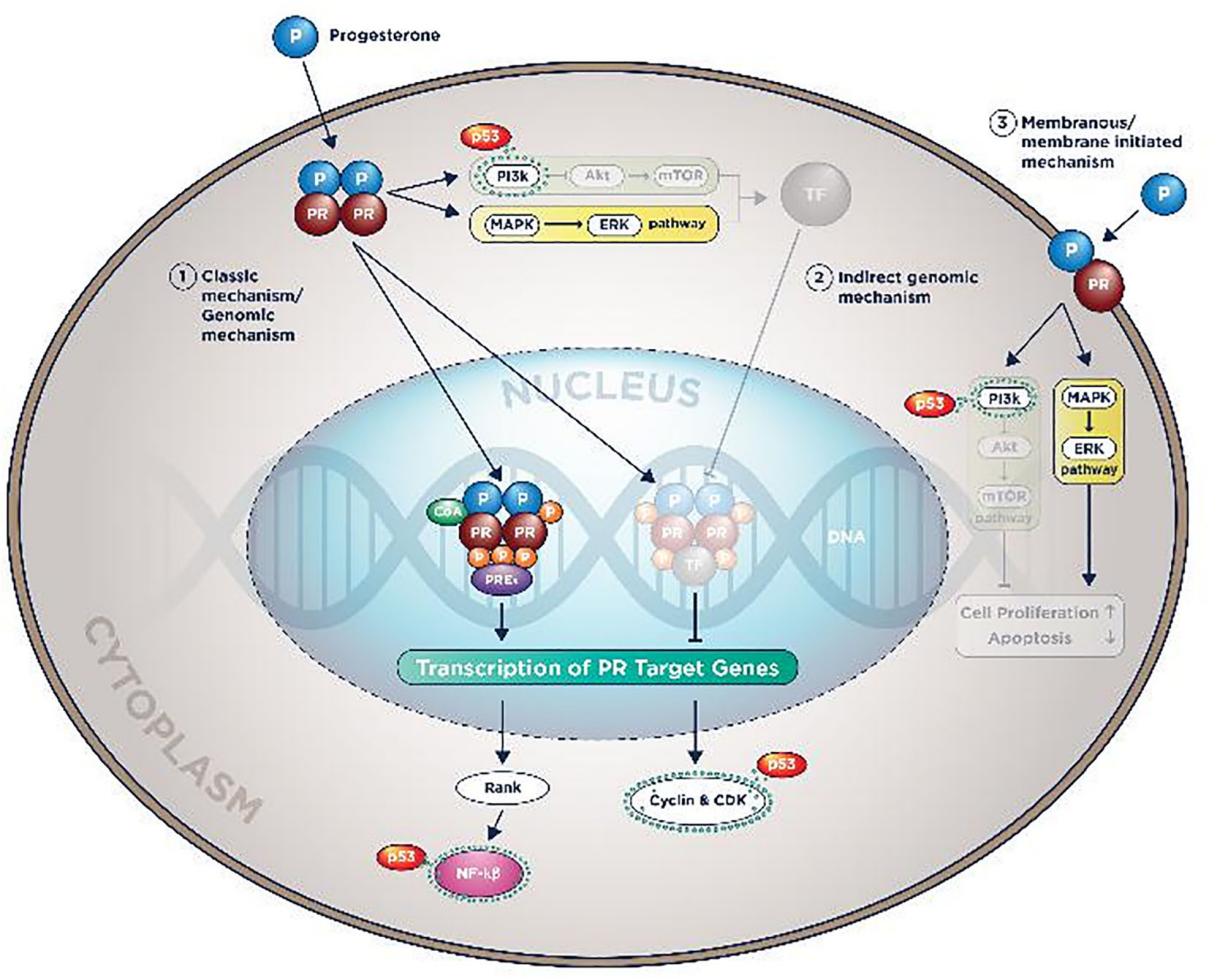
Plausible p53 *wild-type* roles in reducing endocrine therapy resistance by prohibiting PR molecular actions.

A summary of all p53 works in ER-α and PR molecular actions can be seen in Figures 3 and 4.

Distinguished studies also showed us that numerous studies of non-coding RNA especially miRNA play important roles in ET resistance, such as miRNA such as miRNA-1972, miRNA-375 and miRNA-221. ^52^ ^,^ ^88^

However there are still no clear depiction of p53 roles in ET resistance caused by miRNA involvement. It is due to the complexity of the network of both parties, in which miRNA could regulate p53 level and function and vice versa mutated p53 could regulate the miRNA expression and modulate miRNA biology activities due to its gain-of-function properties. ^89^

## Limitation of p53 usage as a predictive biomarker in luminal breast cancer

Although p53 is prominently correlated with poorer clinical features such as a high proliferation index and higher grade and stadium, its usage in Luminal BC is still arguably limited. ^90^ One possible cause is that the presence of ER seems to suppress the p53 mutation itself. ^91^ From the epidemiological point of view, p53 mutation is more frequently found in HER-2 enriched group and the Triple Negative BC group rather than Luminal BC. However, the p53 mutation, when found in Luminal BC, is not without importance. In fact study by Lee *et al.* 2013 from 7739 patients showed us that p53 mutation is correlated with a higher proliferative index such as Ki-67 in Luminal A BC, and when combined, they affect the long-term survival of the patients. ^92^

Since it was found 40 years ago, the p53 protein has been published in numerous cancer studies using protein detection or genetic testing. These factors and the versatility of p53 function in cells make a test for p53 widely known and readily available in most laboratories. Therefore p53 is an ideal predictor to be chosen. ^34^

p53 protein accumulation is easily detected by immunohistochemistry (IHC) as a surrogate marker of its mutation. However, p53 immunohistochemistry testing in BC could present and correlates either with or without favorable gene mutations tested, further affecting its capability as a biomarker in BC. ^91^ It is an obstacle that is also frequently found in other cancers, such as ovarian and gastric cancer. ^93^ ^,^ ^94^

No clear cut-off of p53 positivity in IHC assay also has been noted as the cause and affecting p53 usage as a predictive biomarker in BC. ^95^ Study by Kikuchi *et al.* 2013 tried to address this cut-off issue found that when we set the cut-off of p53 immunoreactivity into ≥50%, then it could be helpful to predict clinical behavior in Luminal Breast Cancer, especially Luminal B type (p<0.0001). ^96^ This finding is also confirmed by another epidemiological study of 7226 patients by Abubakar *et al.* in 2019. ^30^

Furthermore, p53 protein accumulation has been a limitation as a predictor due to many p53 protein isoforms formed within the tissue. These isoforms are many, and each is said to have its roles in molecular effects in cancer cells. ^27^ ^,^ ^91^ ^,^ ^97^ ^,^ ^98^ This limitation has been countered with the suggestion of genetic testing such as PAM50 or Mammaprint that would replace the p53 protein accumulation testing. Although it has been deemed more accurate than the IHC assay, genetic testing is expensive and not readily available in most laboratories, becoming the deterrent factor for choosing this testing in a clinical setting. ^99^

## Future perspective

Endocrine therapy is a very beneficial therapy for Luminal BC patients. Its usage will decrease the 15-year mortality rate to 30-40%; consequently, its resistance will pose patients with a dismal prognosis. As mentioned above, an established predictive biomarker will help clinicians to identify which patients will not have ET benefits in the first place. Therefore their adjuvant therapy should be changed to other modalities to reduce recurrence and increase the overall survival rate. ^2^ In future perspective, a predictive biomarker that could anticipate ET is undoubtedly needed for developing personalized and tailored therapy for Luminal BC patients. ^2^ ^,^ ^26^

Developing and planning studies to identify such biomarkers is not easy since the endocrine therapy resistance theories mentioned before are complex. Estrogen metabolism in premenopausal and postmenopausal women are also different; hence the endocrine therapy given is different; therefore, these groups cannot be investigated together. ^5^ ^,^ ^44^ ^,^ ^100^ ^,^ ^101^ With the previous reasons mentioned, a meticulously planned study embedded in RCT with carefully chosen patients and prospective analysis probably is best to identify such biomarker, explained very well in the seminal study by Beelen *et al*. ^3^

Additionally, p53 mutation could occur as early as the pre-carcinogenesis period, in the early stage of BC, and in late/metastatic disease. ^19^ Consequently, it will be compulsory to test the p53 mutation along the course of the disease and observe whether it correlates with ET resistance later.

p53 is also known to have particular effects in each type of breast cancer (luminal A/B, with or without HER2 positive status) due to BC heterogeneity. ^102^ Several studies have been made to address this issue and concluded that Luminal B breast cancer is the most probable BC group in which p53 mutation could be helpful as a predictive biomarker to predict ET resistance occurrence. ^92^ ^,^ ^102^ This fact is also supported by epidemiological data that showed p53 mutation was found in higher in Luminal B BC compared to Luminal A BC. ^29^ ^,^ ^30^ ^,^ ^103^

Another exciting development is Neoadjuvant Endocrine Therapy (NET), which is currently being studied in a clinical trial, and reportedly has advantages in downstaging and increasing Breast Conserving Therapy (BCT) success. ^104^ In the future, NET could reduce hospital stays for Luminal BC patients and outreach the undertreated patients group. When patients cannot go to the hospital for various reasons, endocrine therapy could provide more accessible and comfortable neo-adjuvant treatment than chemotherapy or radiotherapy. ^104^ Therefore, the need to find such a predictive biomarker becomes more pressing and indispensable, and we have to explore p53 mutation as a plausible biomarker.

## Conclusion

p53 is an important biomarker to be considered an ideal candidate to anticipate ET resistance in the future. Its role within pathways involved in the ER and PR molecular mechanisms is paramount and cannot be ignored. Its limitation as a predictor could be countered using proper genetic testing rather than protein marker. Well-planned studies will be a prerequisite to concluding whether p53 is truly useful as a predictive biomarker for ET resistance in Luminal BC patients, especially the Luminal B group, with an adequate observation period.

## Data availability

No data are associated with this article.

## Authors’ contributions

FH worked on Conceptualization, Data Curation, Formal Analysis, Funding Acquisition, Investigation, Methodology, Resources, Software, Visualization, Writing – Original Draft Preparation. YA involved in Conceptualization, Data Curation, Formal Analysis, Supervision, Validation, Writing – Review & Editing. SS involved in Project Administration, Resources, Software, Supervision, Writing – Review & Editing. FH wrote the draft of the article, YA and SS helped with final manuscript preparation. BH involved in Conceptualization, Project Administration, Software, Supervision, Validation, Writing – Review & Editing. All figures and animations are original to this manuscript, composed by FH and approved by YA, SS and BH. All authors read and approved the final manuscript.

## References

[1] Fontes-Sousa M Amorim M Salta S : Predicting resistance to endocrine therapy in breast cancer: It’s time for epigenetic biomarkers (Review). *Oncol. Rep.* 2019;41(3):1431–1438. 10.3892/or.2019.6967 30664168

[2] Krauss K Stickeler E : Endocrine Therapy in Early Breast Cancer. *Breast Care.* 2020;15(4):337–346. 10.1159/000509362 32982643 PMC7490651

[3] Beelen K Zwart W Linn SC : Can predictive biomarkers in breast cancer guide adjuvant endocrine therapy? *Nat. Rev. Clin. Oncol.* 2012;9(9):529–541. 10.1038/nrclinonc.2012.121 22825374

[4] Zattari E Leporati R Ligorio F : Hormone Receptor Loss in Breast Cancer: Molecular Mechanisms, Clinical Settings, and Therapeutic Implications. *Cells.* 2020;9(2644):1–23. 10.3390/cells9122644 PMC7764472 33316954

[5] Haque MM Desai KV : *Pathways to Endocrine Therapy Resistance in Breast Cancer. Vol. 10, Frontiers in Endocrinology.* Frontiers Media S.A.;2019. 10.3389/fendo.2019.00573 PMC6712962 31496995

[6] De Santo I McCartney A Malorni L : The emerging role of esr1 mutations in luminal breast cancer as a prognostic and predictive biomarker of response to endocrine therapy. *Cancers (Basel).* 2019;11(12):1–15. 10.3390/cancers11121894 PMC6966519 31795152

[7] Fan P Craig JV : New insights into acquired endocrine resistance of breast cancer. *Cancer Drug Resist.* 2019;2(2):198–209. 10.20517/cdr.2019.13 31815253 PMC6897388

[8] Khongthong P Roseweir AK Edwards J : The NF-KB pathway and endocrine therapy resistance in breast cancer. *Endocr. Relat. Cancer.* 2019;26(6):R369–R380. 10.1530/ERC-19-0087 32013374

[9] Belachew EB Sewasew DT : Molecular Mechanisms of Endocrine Resistance in Estrogen-Positive Breast Cancer. *Front Endocrinol (Lausanne).* 2021;12(March):1–11. 10.3389/fendo.2021.599586 PMC8030661 33841325

[10] Gomes I Almeida BP de Dâmaso S : Expression of receptor activator of NFkB (RANK) drives stemness and resistance to therapy in ER+HER2- breast cancer. *Oncotarget.* 2020;11(19):1714–1728. 10.18632/oncotarget.27576 Reference Source 32477461 PMC7233807

[11] Araki K Miyoshi Y : Mechanism of resistance to endocrine therapy in breast cancer: the important role of PI3K/Akt/mTOR in estrogen receptor-positive, HER2-negative breast cancer. *Breast Cancer.* 2018;25(4):392–401. 10.1007/s12282-017-0812-x 29086897

[12] Presti D Quaqarini E : The PI3K/AKT/mTOR and CDK4/6 Pathways in Endocrine Resistant HR+/HER2− Metastatic Breast Cancer: Biological Mechanisms and New Treatments. *Cancer Res.* 2019;11(9):1–20. 10.3390/cancers11091242 Reference Source PMC6770492 31450618

[13] Weiss RH : p21Waf1/Cip1 as a therapeutic target in breast and other cancers. *Cancer Cell.* 2003;4(December):425–429. 10.1016/S1535-6108(03)00308-8 14706334

[14] Karimian A Ahmadi Y Yousefi B : Multiple functions of p21 in cell cycle, apoptosis and transcriptional regulation after DNA damage. *DNA Repair (Amst).* 2016;42:63–71. 10.1016/j.dnarep.2016.04.008 27156098

[15] Cenciarini ME Proietti CJ : Molecular mechanisms underlying progesterone receptor action in breast cancer: Insights into cell proliferation and stem cell regulation. *Steroids.* 2019;152(June):108503. 10.1016/j.steroids.2019.108503 31562879

[16] Rastelli F Crispino S : Factors predictive of response to hormone therapy in breast cancer. *Tumori.* 2008;94(3):370–383. 10.1177/030089160809400314 18705406

[17] Al Saleh S Sharaf LH Luqmani YA : Signalling pathways involved in endocrine resistance in breast cancer and associations with epithelial to mesenchymal transition (Review). *Int. J. Oncol.* 2011;38(5):1197–1217. 10.3892/ijo.2011.942 21318221

[18] Pearson A Proszek P Pascual J : Inactivating NF1 mutations are enriched in advanced breast cancer and contribute to endocrine therapy resistance. *Clin. Cancer Res.* 2020;26(3):608–622. 10.1158/1078-0432.CCR-18-4044 31591187

[19] Bertucci F Ng CKY Patsouris A : Genomic characterization of metastatic breast cancers. *Nature.* 2019;569(7757):560–564. 10.1038/s41586-019-1056-z 31118521

[20] Velloso FJ Bianco AFR Farias JO : The crossroads of breast cancer progression: Insights into the modulation of major signaling pathways. *Onco. Targets. Ther.* 2017;10:5491–5524. 10.2147/OTT.S142154 29200866 PMC5701508

[21] Hanker AB Sudhan DR Arteaga CL : Overcoming Endocrine Resistance in Breast Cancer. *Cancer Cell.* 2020;37(4):496–513. 10.1016/j.ccell.2020.03.009 32289273 PMC7169993

[22] Mills JN Rutkovsky AC Giordano A : Mechanisms of resistance in estrogen receptor positive breast cancer: overcoming resistance to tamoxifen/aromatase inhibitors. *Curr. Opin. Pharmacol.* 2018;41:59–65. 10.1016/j.coph.2018.04.009 29719270 PMC6454890

[23] Brufsky AM Dickler MN : Estrogen Receptor-Positive Breast Cancer: Exploiting Signaling Pathways Implicated in Endocrine Resistance. *Oncologist.* 2018;23(5):528–539. 10.1634/theoncologist.2017-0423 29352052 PMC5947450

[24] Kingston B Cutts RJ Bye H : Genomic profile of advanced breast cancer in circulating tumour DNA. *Nat. Commun.* 2021;12(1):2423. 10.1038/s41467-021-22605-2 33893289 PMC8065112

[25] Wang X Fang Y Sun W : Endocrinotherapy resistance of prostate and breast cancer: Importance of the NF–κB pathway (Review). *Int. J. Oncol.* 2020;56(5):1064–1074. 10.3892/ijo.2020.4990 32319568

[26] Cardoso F Paluch-Shimon S Senkus E : 5th ESO-ESMO international consensus guidelines for advanced breast cancer (ABC 5). *Ann. Oncol.* 2020;31(12):1623–1649. 10.1016/j.annonc.2020.09.010 32979513 PMC7510449

[27] Duffy MJ Synnott NC Crown J : Mutant p53 in breast cancer: potential as a therapeutic target and biomarker. *Breast Cancer Res. Treat.* 2018;170(2):213–219. 10.1007/s10549-018-4753-7 29564741

[28] Silwal-Pandit L Vollan HKM Chin SF : TP53 mutation spectrum in breast cancer is subtype specific and has distinct prognostic relevance. *Clin. Cancer Res.* 2014;20(13):3569–3580. 10.1158/1078-0432.CCR-13-2943 24803582

[29] Nik-Zainal S Davies H Staaf J : Landscape of somatic mutations in 560 breast cancer whole genome sequences. 2016;534(7605):47–54. 10.1038/nature17676 PMC4910866 27135926

[30] Abubakar M Guo C Koka H : Clinicopathological and epidemiological significance of breast cancer subtype reclassification based on p53 immunohistochemical expression. *npj Breast Cancer.* 2019;5(1):1–9. 10.1038/s41523-019-0117-7 31372496 PMC6658470

[31] Yamashita H Toyama T Nishio M : P53 Protein Accumulation Predicts Resistance To Endocrine Therapy and Decreased Post-Relapse Survival in Metastatic Breast Cancer. *Breast Cancer Res.* 2006;8(4):1–8. 10.1186/bcr1536 PMC1779473 16869955

[32] Jia XQ Hong Q Cheng JY : Accumulation of p53 is prognostic for aromatase inhibitor resistance in early-stage postmenopausal patients with ER-positive breast cancer. *Onco. Targets. Ther.* 2015;8:549–555. 10.2147/OTT.S76879 25767399 PMC4354449

[33] Gasco M Shami S Crook T : The p53 pathway in breast cancer. *Breast Cancer Res.* 2002;4(2):70–76. 10.1186/bcr426 11879567 PMC138723

[34] Levine AJ : P53 and the immune response: 40 years of exploration—a plan for the future. *Int. J. Mol. Sci.* 2020;21(2). 10.3390/ijms21020541 31952115 PMC7013403

[35] Muñoz-Fontela C Mandinova A Aaronson SA : Emerging roles of p53 and other tumour-suppressor genes in immune regulation. *Nat. Rev. Immunol.* 2016;16(12):741–750. 10.1038/nri.2016.99 27667712 PMC5325695

[36] Luque-Bolivar A Pérez-Mora E Villegas VE : Resistance and overcoming resistance in breast cancer. *Breast Cancer Targets Ther.* 2020;12:211–229. 10.2147/BCTT.S270799 33204149 PMC7666993

[37] Liu Y Leslie PL Zhang Y : Life and Death Decision-Making by p53 and Implications for Cancer Immunotherapy. *Trends Cancer. Cell Press.* 2021;7:226–239. 10.1016/j.trecan.2020.10.005 33199193 PMC7889652

[38] Yamamoto M Hosoda M Nakano K : P53 accumulation is a strong predictor of recurrence in estrogen receptor-positive breast cancer patients treated with aromatase inhibitors. *Cancer Sci.* 2014;105(1):81–88. 10.1111/cas.12302 24118529 PMC4317887

[39] Kingston B Cutts RJ Bye H : Genomic profile of advanced breast cancer in circulating tumour DNA. *Nat. Commun.* 2021;12(1). 10.1038/s41467-021-22605-2 PMC8065112 33893289

[40] Hynes NE Watson CJ : Mammary gland growth factors: roles in normal development and in cancer. *Cold Spring Harb. Perspect. Biol.* 2010;2(8):1–18. 10.1101/cshperspect.a003186 PMC2908768 20554705

[41] Russo J Russo H : I. the Role of Estrogen in the Initation of Brest Cancer. *J. Steroid Biochem. Mol. Biol.* 2007;102:89–96. 10.1016/j.jsbmb.2006.09.004 Reference Source PMC1832080 17113977

[42] Yaghjyan L Colditz GA : Estrogens in the breast tissue: A systematic review. *Cancer Causes Control.* 2011;22(4):529–540. 10.1007/s10552-011-9729-4 21286801 PMC3652894

[43] Padrão NA Mayayo-Peralta I Zwart W : Targeting mutated estrogen receptor alpha: Rediscovering old and identifying new therapeutic strategies in metastatic breast cancer treatment. *Curr. Opin. Endocr. Metab. Res.* 2020;15:43–48. 10.1016/j.coemr.2020.10.008

[44] Vrtačnik P Ostanek B Mencej-Bedrač S : The many faces of estrogen signaling. *Biochem. Med.* 2014;24(3):329–342. 10.11613/BM.2014.035 25351351 PMC4210253

[45] Szostakowska M Trębińska-Stryjewska A Grzybowska EA : Resistance to endocrine therapy in breast cancer: molecular mechanisms and future goals. *Breast Cancer Res. Treat.* 2019;173(3):489–497. 10.1007/s10549-018-5023-4 30382472 PMC6394602

[46] Lewis JS Jordan VC : Selective estrogen receptor modulators (SERMs): Mechanisms of anticarcinogenesis and drug resistance. *Mutat. Res. - Fundam. Mol. Mech. Mutagen.* 2005;591(1–2):247–263. 10.1016/j.mrfmmm.2005.02.028 16083919

[47] Heger Z Zitka O Krizkova S : Receptor Complex Binding To Estrogen Response. 2013(August 2015). 24362104

[48] Dalvai M Bystricky K : Cell cycle and anti-estrogen effects synergize to regulate cell proliferation and er target gene expression. *PLoS One.* 2010;5(6):1–9. 10.1371/journal.pone.0011011 PMC2882356 20543978

[49] Yang W Schwartz GN Marotti JD : Estrogen receptor alpha drives mTORC1 inhibitor-induced feedback activation of PI3K/AKT in ER+ breast cancer. *Oncotarget.* 2018;9(10):8810–8822. 10.18632/oncotarget.24256 29507656 PMC5823630

[50] Miricescu D Totan A Stanescu-Spinu II : PI3K/AKT/mTOR signaling pathway in breast cancer: From molecular landscape to clinical aspects. *Int. J. Mol. Sci.* 2021;22(1):1–24. 10.3390/ijms22010173 PMC7796017 33375317

[51] Oida K Matsuda A Jung K : Nuclear factor-Ä̧B plays a critical role in both intrinsic and acquired resistance against endocrine therapy in human breast cancer cells. *Sci. Rep.* 2014;4:1–8. 10.1038/srep04057 PMC3925966 24531845

[52] Behringer A Stoimenovski D Porsch M : Relationship of micro-RNA, mRNA and eIF Expression in Tamoxifen-Adapted MCF-7 Breast Cancer Cells: Impact of miR-1972 on Gene Expression. *Proliferation and Migration Biomolecules.* 2022;12(7). 10.3390/biom12070916 PMC9312698 35883472

[53] Bui T Gu Y Ancot F : Emergence of β1 integrin-deficient breast tumours from dormancy involves both inactivation of p53 and generation of a permissive tumour microenvironment. *Oncogene.* 2022;41(4):527–537. 34782719 10.1038/s41388-021-02107-7 PMC8782722

[54] Alves CL Elias D Lyng MB : SNAI2 upregulation is associated with an aggressive phenotype in fulvestrant- resistant breast cancer cells and is an indicator of poor response to endocrine therapy in estrogen receptor-positive metastatic breast cancer. 2018;1–12. 10.1186/s13058-018-0988-9 PMC6009053 29921289

[55] Kuhl H Schneider HPG : Progesterone - Promoter or inhibitor of breast cancer. *Climacteric.* 2013;16(S1):54–68. 10.3109/13697137.2013.768806 23336704

[56] Trabert B Sherman ME Nagarajan Kannan FZS : Progesterone and breast cancer. *Endocr. Rev.* 2020;41(2):320–344. 10.1210/endrev/bnz001 31512725 PMC7156851

[57] Cui X Schiff R Arpino G : Biology of progesterone receptor loss in breast cancer and its implications for endocrine therapy. *J .Clin. Oncol.* 2005;23(30):7721–7735. 16234531 10.1200/JCO.2005.09.004

[58] Fendrick JL Raafat AM Haslam SZ : Mammary Gland Growth and Development from the Postnatal Period to Postmenopause: Ovarian Steroid Receptor Ontogeny and Regulation in the Mouse. *J. Mammary Gland Biol. Neoplasia.* 1998;3(1):7–22. 10.1023/A:1018766000275 10819501

[59] Cline JM Soderqvist G Von Schoultz E : Effects of hormone replacement therapy on the mammary gland of surgically postmenopausal cynomolgus macaques. *Am. J. Obstet. Gynecol.* 1996;174(1):93–100. 10.1016/S0002-9378(96)70379-4 8572040

[60] Joshi PA Goodwin PJ Khokha R : Progesterone exposure and breast cancer risk: Understanding the biological roots. *JAMA Oncol.* 2015;1(3):283–286. 10.1001/jamaoncol.2015.0512 26181171

[61] Obr AE Edwards DP : The biology of progesterone receptor in the normal mammary gland and in breast cancer. *Mol. Cell. Endocrinol.* 2012;357(1–2):4–17. 10.1016/j.mce.2011.10.030 22193050 PMC3318965

[62] Diep CH Ahrendt H Lange CA : Progesterone induces progesterone receptor gene (PGR) expression via rapid activation of protein kinase pathways required for cooperative estrogen receptor alpha (ER) and progesterone receptor (PR) genomic action at ER/PR target genes. *Steroids.* 2016;114(114):48–58. 10.1016/j.steroids.2016.09.004 Reference Source 27641443 PMC5068826

[63] Li X O’Malley BW : Unfolding the action of progesterone receptors. *J. Biol. Chem.* 2003;278(41):39261–39264. 10.1074/jbc.R300024200 12893816

[64] Chen J : The cell-cycle arrest and apoptotic and progression. *Cold Spring Harb. Perspect. Biol.* 2016:1–16. 10.1101/cshperspect.a026104 PMC4772082 26931810

[65] Peurala E Koivunen P Haapasaari KM : The prognostic significance and value of cyclin D1, CDK4 and p16 in human breast cancer. *Breast Cancer Res.* 2013;15(1):R5. 10.1186/bcr3376 Reference Source 23336272 PMC3672746

[66] Cardoso F Kyriakides S Ohno S : Early breast cancer-ESMO Clinical Practice Guidelines for Diagnosis, Treatment and Follow-up. 2019 [cited 2021 May 24]. Reference Source 10.1093/annonc/mdz189 31236598

[67] Wang L Han H Dong L : Function of p21 and its therapeutic effects in esophageal cancer (Review). *Oncol. Lett.* 2021;21(2):1–7. 33552255 10.3892/ol.2020.12397 PMC7798030

[68] Kulaberoglu Y Gundogdu R Hergovich A : The Role of p53/p21/p16 in DNA-Damage Signaling and DNA Repair. *Genome Stability: From Virus to Human Application.* 2016;243–256p. Elsevier Inc. 10.1016/B978-0-12-803309-8.00015-X

[69] Alzahrani AS : PI3K/Akt/mTOR inhibitors in cancer: At the bench and bedside. *Semin Cancer Biol [Internet].* 2019;April:0–1. 10.1016/j.semcancer.2019.07.009 31323288

[70] Osborne CK Schiff R : Mechanisms of endocrine resistance in breast cancer. *Annu. Rev. Med.* 2011;62:233–247. 10.1146/annurev-med-070909-182917 20887199 PMC3656649

[71] Álvarez-Garcia V Tawil Y Wise HM : Mechanisms of PTEN loss in cancer: It’s all about diversity. *Semin. Cancer Biol.* 2019;59(January):66–79. 10.1016/j.semcancer.2019.02.001 30738865

[72] Stambolic V MacPherson D Sas D : Regulation of PTEN transcription by p53. *Mol. Cell.* 2001;8(2):317–325. 10.1016/S1097-2765(01)00323-9 11545734

[73] Jung SH Hwang HJ Kang D : mTOR kinase leads to PTEN-loss-induced cellular senescence by phosphorylating p53. *Oncogene.* 2019;38(10):1639–1650. 10.1038/s41388-018-0521-8 30337688 PMC6755978

[74] Galon J Bruni D : Approaches to treat immune hot, altered and cold tumours with combination immunotherapies. *Nat. Rev. Drug Discov.* 2019;18(3):197–218. 30610226 10.1038/s41573-018-0007-y

[75] Goldberg J Pastorello RG Vallius T :2021; *The Immunology of Hormone Receptor Positive Breast Cancer* , vol.12. Frontiers Media SA: Frontiers in Immunology. 10.3389/fimmu.2021.674192 PMC8202289 34135901

[76] Anurag M Zhu M Huang C : Immune Checkpoint Profiles in Luminal B Breast Cancer (Alliance). *J Natl Cancer Inst.* 2020;112(7). 10.1093/jnci/djz213 PMC7805027 31665365

[77] Stanton SE Adams S Disis ML : Variation in the Incidence and Magnitude of Tumor-Infiltrating Lymphocytes in Breast Cancer Subtypes: A Systematic Review. *JAMA Oncol.* 2016;2(10):1354–1360. 27355489 10.1001/jamaoncol.2016.1061

[78] Zhou Y Eppenberger-Castori S Eppenberger U : The NFκB pathway and endocrine-resistant breast cancer. *Endocr Relat Cancer.* 2005;12(SUPPL. 1):37–46. 10.1677/erc.1.00977 16113098

[79] Rani A Stebbing J Giamas G : Endocrine resistance in hormone receptor positive breast cancer–from mechanism to therapy. *Front Endocrinol (Lausanne).* 2019;10(MAY). 10.3389/fendo.2019.00245 PMC6543000 31178825

[80] Sobral-Leite M Salomon I Opdam M : Cancer-immune interactions in ER-positive breast cancers: PI3K pathway alterations and tumor-infiltrating lymphocytes. *Breast Cancer Res.* 2019;21(1):1–12. 31391067 10.1186/s13058-019-1176-2 PMC6686400

[81] Pal S Bhattacharjee A Ali A : Chronic inflammation and cancer: Potential chemoprevention through nuclear factor kappa B and p53 mutual antagonism. *J Inflamm. (United Kingdom).* 2014;11(1):23. 10.1186/1476-9255-11-23 PMC4142057 25152696

[82] Dolcet X Llobet D Pallares J : NF-kB in development and progression of human cancer. *Virchows Arch.* 2005;446(5):475–482. 10.1007/s00428-005-1264-9 15856292

[83] Nakshatri H Bhat-Nakshatri P Martin DA : Constitutive activation of NF-kappaB during progression of breast cancer to hormone-independent growth. *Mol. Cell. Biol.* 1997;17(7):3629–3639. 10.1128/MCB.17.7.3629 9199297 PMC232215

[84] Wang X Belguise K O’Neill CF : RelB NF-κB Represses Estrogen Receptor α Expression via Induction of the Zinc Finger Protein Blimp1. *Mol. Cell. Biol.* 2009;29(14):3832–3844. 10.1128/MCB.00032-09 19433448 PMC2704748

[85] Thomasova D Mulay SR Bruns H : p53-independent roles of MDM2 in NF-κB signaling: Implications for cancer therapy, wound healing, and autoimmune diseases. *Neoplasia (United States).* 2012;14(12):1097–1101. 10.1593/neo.121534 23308042 PMC3540936

[86] Rooks MG Garrett W : p53 Mutations and Inflammation-Associated Cancer Are Linked through TNF Signaling. *Mol. Cell.* 2014;56(5):611–612. 10.1016/j.molcel.2014.11.018 Reference Source 25479634 PMC6333310

[87] Mercogliano MF Bruni S Elizalde PV : Tumor Necrosis Factor α Blockade: An Opportunity to Tackle Breast Cancer. *Front. Oncol.* 2020;10(April). 10.3389/fonc.2020.00584 32391269 PMC7189060

[88] Amiruddin A Massi MN Islam AA : microRNA-221 and tamoxifen resistance in luminal-subtype breast cancer patients: A case-control study. Ann. Med. Surg. [Internet]. 2022;73(October 2021): 103092. 10.1016/j.amsu.2021.103092 PMC8767262 35079352

[89] Liu J Zhang C Zhao Y : MicroRNA Control of p53. *J Cell Biochem.* 2017;118(1):7–14. 27216701 10.1002/jcb.25609

[90] Sadighi S Zokaasadi M Kasaeian A : The effect of immunohistochemically detected p53 accumulation in prognosis of breast cancer; a retrospective survey of outcome. *PLoS One.* 2017;12(8):1–10. 10.1371/journal.pone.0182444 PMC5542560 28771563

[91] Coates AS Millar EKA O’Toole SA : Prognostic interaction between expression of p53 and estrogen receptor in patients with node-negative breast cancer: Results from IBCSG Trials VIII and IX. *Breast Cancer Res.* 2012;14(6):R143. 10.1186/bcr3348 23127292 PMC4053129

[92] Lee SK Bae SY Lee JH : Distinguishing Low-Risk Luminal A Breast Cancer Subtypes with Ki-67 and p53 Is More Predictive of Long-Term. *Survival.* 2015;10:1–14. 10.1371/journal.pone.0124658 PMC4524613 26241661

[93] Köbel M Piskorz AM Lee S : Optimized p53 immunohistochemistry is an accurate predictor of TP53 mutation in ovarian carcinoma. *J. Pathol. Clin. Res.* 2016;2(4):247–258. 10.1002/cjp2.53 27840695 PMC5091634

[94] Fondevila C Metges JP Fuster J : p53 and VEGF expression are independent predictors of tumour recurrence and survival following curative resection of gastric cancer. *Br. J. Cancer.* 2004;90(1):206–215. 10.1038/sj.bjc.6601455 14710231 PMC2395306

[95] Milićević Z Bajić V Živković L : Identification of p53 and its isoforms in human breast carcinoma cells. *Sci. World J.* 2014;2014:1–10. 10.1155/2014/618698 24511294 PMC3913390

[96] Kikuchi S Nishimura R Osako T : Definition of p53 overexpression and its association with the clinicopathological features in luminal/HER2-negative breast cancer. *Anticancer Res.* 2013;33(9):3891–3897. 24023325

[97] Bischof K Knappskog S Hjelle SM : Influence of p53 Isoform Expression on Survival in High-Grade Serous Ovarian Cancers. *Sci. Rep.* 2019;9(1):1–11. 30918304 10.1038/s41598-019-41706-z PMC6437169

[98] Avery-kiejda KA Morten B Wong-brown MW : The relative mRNA expression of p53 isoforms in breast cancer is associated with clinical features and outcome. 2014;35(3):586–596. 10.1093/carcin/bgt411 24336193

[99] Alfarsi L Johnston S Liu DX : Current issues with luminal subtype classification in terms of prediction of benefit from endocrine therapy in early breast cancer. *Histopathology.* 2018;73(4):545–558. 10.1111/his.13523 29603357

[100] Segovia-Mendoza M Morales-Montor J : Immune tumor microenvironment in breast cancer and the participation of estrogens and its receptors into cancer physiopathology. *Front. Immunol.* 2019;10(MAR):1–16. 10.3389/fimmu.2019.00348 30881360 PMC6407672

[101] Tower H Ruppert M Britt K : The Immune Microenvironment of Breast Cancer Progression. 2019. Reference Source 10.3390/cancers11091375 PMC6769749 31527531

[102] Ahn SH Kim HJ Han W : Breast Cancer Effect Modification of Hormonal Therapy by p53 Status in Invasive. *Breast Cancer.* 2013;16(4):386–394. 10.4048/jbc.2013.16.4.386 24454460 PMC3893340

[103] Chuangsuwanich T Pongpruttipan T O-charoenrat P : Clinicopathologic features of breast carcinomas classified by biomarkers and correlation with microvessel density and VEGF expression: A study from Thailand. *Asian Pac. J. Cancer Prev.* 2014;15(3):1187–1192. 10.7314/APJCP.2014.15.3.1187 24606439

[104] Madigan LI Dinh P Graham JD : Neoadjuvant endocrine therapy in locally advanced estrogen or progesterone receptor-positive breast cancer: Determining the optimal endocrine agent and treatment duration in postmenopausal women-a literature review and proposed guidelines. *Breast Cancer Res.* 2020;22(1):1–13. 10.1186/s13058-020-01314-6 PMC7370425 32690069

