# Supplementary material for: p53 Mutation as Plausible Predictor for Endocrine Resistance Therapy in Luminal Breast Cancer
Source: F1000Res. 2022 Nov 30;11:330. Originally published 2022 Mar 18. [Version 2] doi: 10.12688/f1000research.108628.2 (PMC9718986; doi:10.12688/f1000research.108628.2)
Supplement: Peer review history

# Peer review: Reviewer response for version 2

**DOI:** 10.5256/f1000research.140986.r157027

**Published:** 2022-12-02

**Authors:** Norbert Nass

**Affiliations:** 1 Department of Pathology, Medical Faculty, Otto-Von-Guericke University Magdeburg, Magdeburg, Germany; 2 Department for Internal Medicine I, Dessau Medical Center, Dessau, Germany

I thank the authors for modifying their manuscript according to my suggestion. This is a stimulating contribution to this field as p53 mutations and abundance have not conclusively been investigated as a potential biomarker for anti-endocrine resistance.

Is the review written in accessible language?

Yes

Are all factual statements correct and adequately supported by citations?

Yes

Are the conclusions drawn appropriate in the context of the current research literature?

Partly

Is the topic of the review discussed comprehensively in the context of the current literature?

Yes

Reviewer Expertise:

Cell Biology, Tamoxifen resistance in breast cancer, protein biosynthesis

I confirm that I have read this submission and believe that I have an appropriate level of expertise to confirm that it is of an acceptable scientific standard.

# Peer review: Reviewer response for version 1

**DOI:** 10.5256/f1000research.120027.r152460

**Published:** 2022-10-17

**Authors:** Norbert Nass

**Affiliations:** 1 Department of Pathology, Medical Faculty, Otto-Von-Guericke University Magdeburg, Magdeburg, Germany; 2 Department for Internal Medicine I, Dessau Medical Center, Dessau, Germany

In this review the authors summarize our current knowledge on a possible function of p53 in the occurrence of resistance towards endocrine therapy in breast cancer.

 The main hypothesis is that p53 might regulate estrogen receptor expression, abundance and signalling. Deregulation or mutation of p53 then causes resistance because the estrogen receptor is a major mediator of endocrine therapy. Therefore p53 should be considered in biomarker studies for anti-endocrine resistance.

 This is an interesting review as p53 mutations and abundance have not conclusively been investigated as a potential biomarker for anti-endocrine resistance. It is also carefully written and illustrated.

 Besides the mechanisms discussed in this manuscript, there is cumulating evidence that the cancer microenvironment as well as non-coding RNAs are also involved in the development of endocrine therapy resistance. I suggest, this should be mentioned here.

 The authors also state that " *Estrogen will form a dimer form, then bind to…*”.  I am not sure if this is true. In my current view, one estrogen molecule binds to one receptor binding domain. Then the estrogen receptor dissociates from e.g. heat shock proteins and forms dimers. Please clarify!

 Later, the authors state “ *The final result of these processes are constant activation of the estrogen receptor target genes*”. I think this not the whole story. Many gene expression studies on e.g. tamoxifen or fulvestrant adapted cell lines show that many apparently estrogen-unrelated genes are also regulated.

 I think it would be worthwhile mentioning that PGR and ESR1 are usually co-expressed and only a few cases are described that express PGR, but not ESR1.

Is the review written in accessible language?

Yes

Are all factual statements correct and adequately supported by citations?

Yes

Are the conclusions drawn appropriate in the context of the current research literature?

Partly

Is the topic of the review discussed comprehensively in the context of the current literature?

Yes

Reviewer Expertise:

Cell Biology, Tamoxifen resistance in breast cancer, protein biosynthesis

I confirm that I have read this submission and believe that I have an appropriate level of expertise to confirm that it is of an acceptable scientific standard, however I have significant reservations, as outlined above.

# Response

**Published:** 2022-11-10

**Authors:** Freda Halim

**Affiliations:** Pelita Harapan University, Indonesia

*"In this review, the authors summarize our current knowledge on a possible function of p53 in the occurrence of resistance towards endocrine therapy in breast cancer.*

*The main hypothesis is that p53 might regulate estrogen receptor expression, abundance, and signaling. Deregulation or mutation of p53 causes resistance because the estrogen receptor is a major mediator of endocrine therapy. Therefore p53 should be considered in biomarker studies for anti-endocrine resistance.*

*This is an interesting review as p53 mutations and abundance have not conclusively been investigated as a potential biomarker for anti-endocrine resistance. It is also carefully written and illustrated."*

**Dear Reviewer, we are grateful for your gracious review of our article, and we attempt to address several issues raised in the comment.**

1. *"Besides the mechanisms discussed in this manuscript, there is cumulating evidence that the cancer microenvironment, as well as non-coding RNAs, are also involved in the development of endocrine therapy resistance. I suggest this should be mentioned here."*
**Answer: **We have already added several postulations about the tumor microenvironment's role in ET resistance and non-coding RNAs.
2. *"The authors also state that "Estrogen will form a dimer form, then bind to…". I am not sure if this is true. Currently, one estrogen molecule binds to one receptor-binding domain. Then the estrogen receptor dissociates from, e.g., heat shock proteins and forms dimers. Please clarify!"*
**Answer:** we acknowledge this error and have already edited the paragraph.
3. *"Later, the authors state, "The final result of these processes is constant activation of the estrogen receptor target genes." I think this is not the whole story. Many gene expression studies on, e.g., tamoxifen or fulvestrant-adapted cell lines show that many apparently estrogen-unrelated genes are also regulated."*
**Answer:** We acknowledged these facts and edited the sentence. We already mentioned that several mechanisms of estrogen-unrelated genes are also regulated in the tamoxifen and fulvestrant-adapted cell lines.
4. *"I think it would be worthwhile mentioning that PGR and ESR1 are usually co-expressed, and only a few cases are described that express PGR but not ESR1."*
**Answer: **We agreed to this comment and already mentioned it in our review, with cited literature.

# Peer review: Reviewer response for version 1

**DOI:** 10.5256/f1000research.120027.r141770

**Published:** 2022-07-12

**Authors:** Didik Setyo Heriyanto

**Affiliations:** 1 Gastroenterology-Hepatology Division of Internal Medicine Department, Faculty of Medicine, Public Health and Nursing, Dr. Sardjito General Hospital, Universitas Gadjah Mada, Yogyakarta, Indonesia

Since 2002, p53-related breast cancer research has been conducted. Regularly, p53 is evaluated for diagnostic and prognostic purposes. In contrast to other solid tumors, breast cancer has a lower incidence of p53 gene mutations. The reviewer suggests that the author also provide updates on recently studied potential biomarkers, particularly those associated with breast cancer. Given that the TP53 mutation is associated with a more aggressive disease and a lower overall survival rate, it is also crucial to identify a potential biomarker that can be tested in the early stages of the disease ^1^.

 This study also states that p53 gene mutation is one of the most common genetic alterations in breast cancer, but its incidence is only 30–35% in Luminal B BC , compared to 70% in Her-2/neu overexpression and 80% in TNBC ^2^. This is also stated as a limitation in the study. The reviewer suggest that the study could also provide additional insight into the recent roles of p53 gene mutation applications as biomarkers in Her-2/neu overexpression and the TNBC group, in which both have higher incidence ^3^.

 It is possible for endocrine resistance to develop during the endocrine therapy or at a later time. While it is possible that the p53 gene mutation is suppressed by the presence of ER, this also means that individuals who are more susceptible to endocrine therapy have a greater chance of doing so naturally. In addition, the reviewer emphasize the importance of determining whether endocrine therapies that interact with ER, such as SERM and SERD, would also affect its suppressive effect on p53 mutation ^4,5^.

 In conclusion, p53 has the potential to become a biomarker for predicting ET resistance in the future, as its participation in the pathways involving the ER and PR molecular mechanisms is essential and cannot be ignored. The author has elaborated on the limitations of the predictability tool. The reviewer concur that additional research is necessary to determine whether p53 is effective as a prognostic biomarker for ET resistance in Luminal BC patients.

Is the review written in accessible language?

Yes

Are all factual statements correct and adequately supported by citations?

Yes

Are the conclusions drawn appropriate in the context of the current research literature?

Yes

Is the topic of the review discussed comprehensively in the context of the current literature?

Yes

Reviewer Expertise:

Airway, Cardiovascular, and Mediastinal Pathology, Gastrointestinal, Liver, and Gallbladder Pathology, General Pathology.

I confirm that I have read this submission and believe that I have an appropriate level of expertise to confirm that it is of an acceptable scientific standard.

## References

[1] : The p53 pathway in breast cancer. *Breast Cancer Res* .2002;4(2) : 10.1186/bcr426 70-6 10.1186/bcr426 11879567PMC138723

[2] : TP53 Mutations and Outcomes in Breast Cancer: Reading beyond the Headlines. *Trends Cancer* .6(2) : 10.1016/j.trecan.2020.01.007 98-110 10.1016/j.trecan.2020.01.007 32061310PMC7931175

[3] : New insight on the biological role of p53 protein as a tumor suppressor: re-evaluation of its clinical significance in triple-negative breast cancer. *Tumour Biol* .2016;37(8) : 10.1007/s13277-016-4990-5 11017-24 10.1007/s13277-016-4990-5 26894602

[4] : p53 protein accumulation predicts resistance to endocrine therapy and decreased post-relapse survival in metastatic breast cancer. *Breast Cancer Res* .2006;8(4) : 10.1186/bcr1536 R48 10.1186/bcr1536 16869955PMC1779473

[5] : Accumulation of p53 is prognostic for aromatase inhibitor resistance in early-stage postmenopausal patients with ER-positive breast cancer. *Onco Targets Ther* .2015;8: 10.2147/OTT.S76879 549-55 10.2147/OTT.S76879 25767399PMC4354449

# Response

**Published:** 2022-07-14

**Authors:** Freda Halim

**Affiliations:** Pelita Harapan University, Indonesia

Dear reviewer, thank you for your comment regarding our article. Indeed we believe p53 is an important marker to be checked in luminal breast cancer because it plays many seminal roles in endocrine resistance pathways. We are delighted that your comment on our work supports this.
